# Critical Analysis of the Causes of In-Hospital Mortality following Colorectal Resection: A Queensland Audit of Surgical Mortality (QASM) Registry Study

**DOI:** 10.1007/s00268-022-06534-9

**Published:** 2022-04-04

**Authors:** Derek Mao, Therese Rey-Conde, John B. North, Raymond P. Lancashire, Sanjeev Naidu, Terence C. Chua

**Affiliations:** 1 Faculty of Medicine and Health, The University of Sydney, Sydney, NSW Australia; 2 Queensland Audit of Surgical Mortality, Royal Australasian College of Surgeons, Brisbane, QLD Australia; 3 Department of General Surgery, Queen Elizabeth II Jubilee Hospital, Brisbane, QLD Australia; 4 School of Medicine, Griffith University, Gold Coast, QLD Australia; 5 Faculty of Medicine, The University of Queensland, Brisbane, QLD Australia

## Abstract

**Background:**

Colorectal resection is a major gastrointestinal operation. Improvements in peri-operative care has led to improved outcomes; however, mortalities still occur. Using data from the Queensland Audit of Surgical Mortality (QASM), this study examines the demographic and clinical characteristics of patients who died in hospital following colorectal resection, and also reports the primary cause of death in this population.

**Methods:**

Patients who died in hospital following colorectal resection in Queensland between January 2010 and December 2020 were identified from the QASM database.

**Results:**

There were 755 patients who died in the 10 year study period. Pre-operatively, the risk of death as subjectively determined by operating surgeons was ‘considerable’ in 397 cases (53.0%) and ‘expected’ in 90 cases (12.0%). The patients had a mean of 2.7 (±1.5) co-morbidities, and a mean American Society of Anaesthesiologists (ASA) score of 3.6 (±0.8). Operations were categorised as emergency in 579 patients (77.2%), with 637 patients (85.0%) requiring post-operative Intensive Care Unit (ICU) support. The primary cause of death was related to a surgical cause in 395 patients (52.7%) and to a medical cause in 355 patients (47.3%). The primary causes of death were advanced surgical pathology (*n*=292, 38.9%), complications from surgery (*n*=103, 13.7%), complications arising from pre-existing medical co-morbidity (*n*=282, 37.6%) or new medical complications unrelated to pre-existing conditions (*n*=73, 9.7%).

**Conclusions:**

Patients who died had significant co-morbidities and often presented emergently with an advanced surgical pathology. Surgical and medical causes of death both contributed equally to the mortality burden.

**Supplementary Information:**

The online version contains supplementary material available at 10.1007/s00268-022-06534-9.

## Introduction

Colorectal resection is a commonly performed operation [1]. Early post-operative mortality following colorectal resection is a rare but recognised event. National colorectal cancer databases report rates of 1% in Australia [2], 3% in the United Kingdom [3] and 0.26–2.39% in the United States [4].

Auditing post-operative deaths against benchmarks such as the national 30-day mortality rate have been commonly recognised as major indicators of both the quality of care provided and hospital performance, with these outcomes recorded in several national quality improvement audits without risk adjustment [5–7]. Despite this, it is recognised that pre-existing medical co-morbidity, surgical complications, and the presenting surgical pathology itself are all factors associated with early post-operative mortality [8–12]. The 30-day mortality rate has also been found to under-estimate the true risk of death following colorectal resection [13, 14].

As data from most post-operative mortality audits are obtained through large-scale coding, they lack detailed information about the circumstances leading up to a post-operative death. Therefore, an understanding of the primary causes of in-hospital mortality following colorectal resection remains a poorly explored area.

The Queensland Audit of Surgical Mortality (QASM) is an independent and peer-reviewed audit of all surgically related deaths occurring in Queensland, Australia [15]. By examining QASM data, we aim to report the demographic and clinical characteristics of patients who died in hospital following colorectal resection, and determine the primary causes of death in this population.

## Material and methods

### The Queensland Audit of Surgical Mortality (QASM)

When a surgically-related in-hospital death occurs, the hospital notifies the QASM, who then prompts the treating surgeon to complete a standardised, 26-item *Surgical Case Form (SCF)* (Appendix S1). This form includes a section where the surgeon is able to outline the course from admission to death in detailed free-text (item 9). The SCF is then de-identified and undergoes ‘first line assessment’ (FLA). During FLA, an anonymous consultant surgeon of the same specialty evaluates the SCF who then determines if no further action is required, or if the case requires ‘second line assessment’ (SLA). SLA is a further evaluation by another surgeon who is provided full access to the patient’s medical records. SLA occurs in approximately 14% of cases, most commonly due to insufficient information in the SCF [16].

### Patient selection

We included all patients who died in hospital following colorectal resection in Queensland between January 2010 and December 2020. We excluded patients who underwent colorectal surgery not involving resection such as colostomy formation, colonic bypass, exploratory operations without resection and rectopexy procedure. Patients undergoing appendicectomy were also excluded.

### Data collection

Data from the SCF were retrospectively collected through the QASM database between January 2021 and May 2021. Chart review was not available. Hospitals were divided by the *Rural, Remote and Metropolitan (RRMA)* Classification (Appendix S2). The resection type, underlying surgical pathology and primary cause of death were each verified or identified with item 9 of the SCF. The primary cause of death was classified as being due to either a surgical cause or medical cause. Surgical causes of death were further classified into death due to an advanced surgical pathology, or death due to complications from surgery. An advanced surgical pathology was defined as a life-threatening surgical disease state where patients may not recover despite best care. Medical causes of death were defined as deaths occurring in the absence of an advanced surgical pathology or major surgical complication; and were further classified into either death due to a complication arising from pre-existing medical co-morbidity, or death due to a new medical complication unrelated to pre-existing conditions.

### Statistical analysis

Quantitative data were calculated on Microsoft Excel (Microsoft, Redmond, WA, USA). Continuous variables were presented as means or medians, with the standard deviation and range specified respectively. Categorial variables were presented as frequencies, with percentages specified. Groups were assessed using *t* test or Chi-squared test as appropriate, with statistically significant results defined at the level of *p* ≤ 0.05.

## Results

### Patient demographics

Seven hundred and fifty-five patients died following colorectal resection between January 1, 2010 and December 31, 2020. The patient demographic information is shown in Table 1. There were 414 males (54.8%) and 341 females (45.2%). The median age of all patients was 76.2 years (range 19.3–99.3 years). There were 305 patients (42.1%) treated in a RRMA Metropolitan 1 hospital; 176 patients (24.3%) treated in a RRMA Metropolitan 2 hospital; 153 patients (21.1%) treated in RRMA 3 hospital; 78 patients (10.8%) treated in a RRMA 4 hospital; and 13 patients (1.8%) treated in a RRMA 5 hospital. There were 617 patients (82.0%) managed in a public hospital and 135 patients (18.0%) managed in a private hospital.

**Table 1:** Patient demographic information

	*N*	%
*Sex*		
Male	414	54.8
Female	341	45.2
*RRMA classification*		
M		
1—capital cities	305	42.1
2—other metropolitan centres (population ≥ 100,000)	176	24.3
R		
1—large rural centres (population 25,000–99,999)	153	21.1
2—small rural centres (population 10,000–24,999)	78	10.8
3—other rural areas (population < 10,000)	13	1.8
Not specified	30	
*Hospital status*		
Public	617	82.0
Private	135	18.0
Not specified	3	
*Risk of death on presentation*		
Minimal	4	0.5
Small	68	9.1
Moderate	190	25.4
Considerable	397	53.0
Expected	90	12.0
Not specified	6	
*Operative urgency*		
Elective	171	22.8
Emergency	579	77.2
Not specified	5	
*ICU requirement*		
Required	637	85.0
Not required	112	15.0
Not specified	6	

*RRMA* Rural, Remote and Metropolitan Areas (RRMA); *M* metropolitan zone; *R* rural zone, *ICU* intensive care unit

**Fig. 1:**
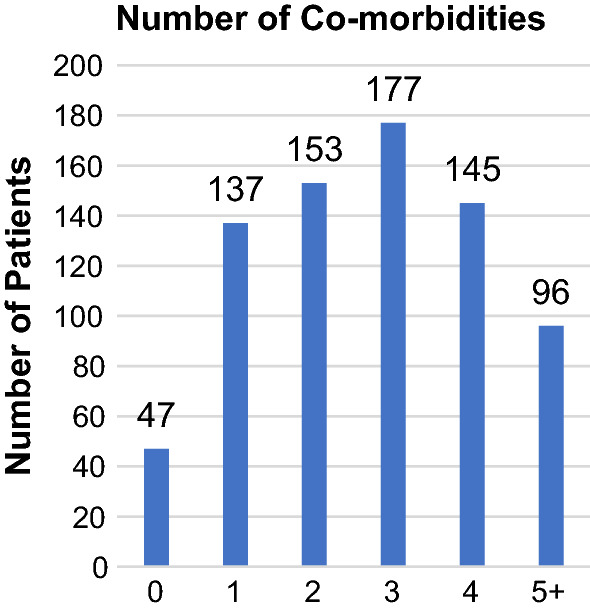
Distribution of the number of co-morbidities per patient

**Fig. 2:**
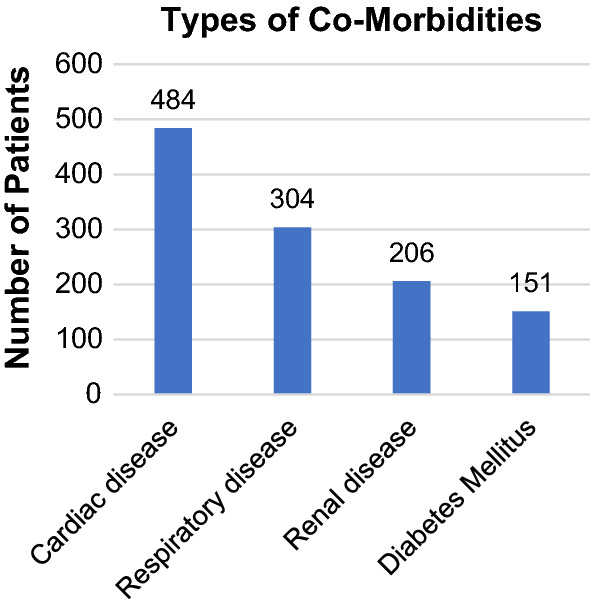
Types of common co-morbidities

**Fig. 3:**
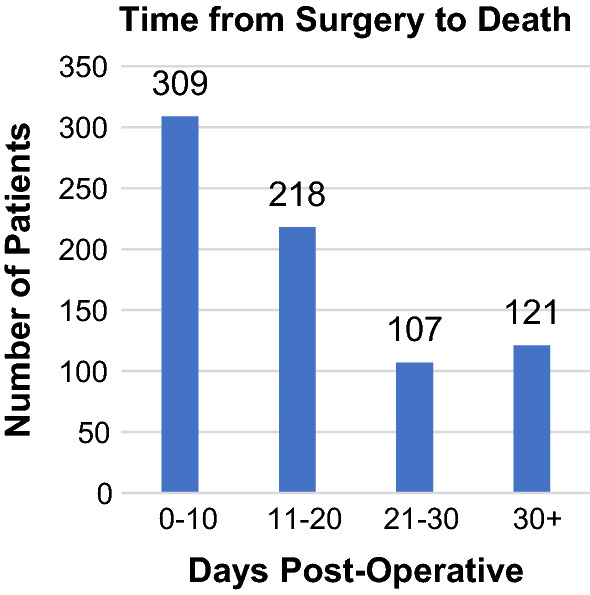
Distribution of the time from surgery to death

The mean number of co-morbidities per patient was 2.7 (±1.5). The distribution and common types of co-morbidities in the cohort are shown in Figs. 1 and 2, respectively. The mean American Society of Anaesthesiologists (ASA) score was 3.6 (±0.8). Operating surgeons determined the risk of death at presentation to be ‘minimal’ in 4 patients (0.5%), ‘small’ in 68 patients (9.1%), ‘moderate’ in 190 patients (25.4%), ‘considerable’ in 397 patients (53.0%) and ‘expected’ in 90 patients (12.0%). Operative urgency was classified as elective in 171 patients (22.8%) and emergency in 579 patients (77.2%). Post-operatively, 637 patients (85.0%) were managed in an Intensive Care Unit (ICU) and 112 patients (15.0%) were managed on a surgical ward. The median time from surgery to death was 17 days (range 0–126 days). The distribution of the time from surgery to death is shown in Fig. 3.

### Resection type

There were 375 right-sided colon resections (49.7%), 284 left-sided colorectal resections (37.6%) and 96 subtotal or total colectomies (12.7%). The two most common resection types in the cohort were right hemicolectomy (*n*=275, 36.4%) and procto-sigmoidectomy (Hartmann’s Procedure) (*n*=137, 18.1%) (Table 2).

**Table 2:** Specific colorectal resection type

	*N*	%
*Right-sided colectomy*		
Right hemicolectomy	275	36.4
Extended right and transverse colectomy	69	9.1
Ileo-caecal resection	22	2.9
Caecectomy	9	1.2
Total	375	49.7
*Left-sided colectomy*		
Procto-sigmoidectomy (Hartmann’s procedure)	137	18.1
Anterior resection	49	6.5
Sigmoid colectomy	39	5.2
Left hemicolectomy	36	4.8
Abdomino-perineal resection	19	2.5
Perineal recto-sigmoidectomy (Altemeier’s procedure)	3	0.4
Perineal proctectomy	1	0.1
Total	284	37.6
*Subtotal and total colectomy*		
Subtotal colectomy	51	6.8
Total colectomy	45	6.0
Total	96	12.7
Total	755	100.0

### Surgical pathology

The underlying surgical pathology were categorised into ‘malignant’ (*n*=305, 40.6%), ‘ischaemic’ (*n*=151, 20.1%), ‘inflammatory or infective’ (*n*=139, 18.5%), ‘obstructive’ (*n*=126, 16.8%) and ‘other’ (*n*=30, 4%) aetiologies. Individually, the three most common diseases implicated in in-hospital mortality were colorectal cancer (*n*=305, 40.6%); ischaemic colitis (*n*=151, 20.1%) and colonic volvulus (*n*=58, 7.7%). The specific surgical pathology of the cohort is shown in Table 3.

**Table 3:** Specific underlying surgical pathology

	*N*	%
*Malignant*		
Colorectal cancer	305	40.6
Total		
*Ischaemic*		20.1
Ischaemic colitis	151	
Total		
*Inflammatory and infective*		
Bowel perforation (NOS)	48	6.4
Diverticular perforation	41	5.5
Stercoral perforation	15	2.0
Colonic abscess (NOS)	11	1.5
Colonic fistula	10	1.3
Infective colitis	10	1.3
Inflammatory colitis	4	0.5
Total	139	18.5
*Obstructive*		
Colonic volvulus	58	7.7
Large bowel obstruction (NOS)	54	7.2
Small bowel obstruction (NOS)	14	1.9
Total	126	16.8
*Other*		
Trauma-related colonic injury	9	1.2
Pancreatitis	7	0.9
Colonic bleeding (NOS)	5	0.7
Rectal prolapse	5	0.7
Appendicitis	4	0.5
Total	30	4.0
Total (all pathologies)	751	100.0
*Unknown*	4	
Total	755	

*NOS* not otherwise specified

### Primary causes of in-hospital mortality

**Fig. 4:**
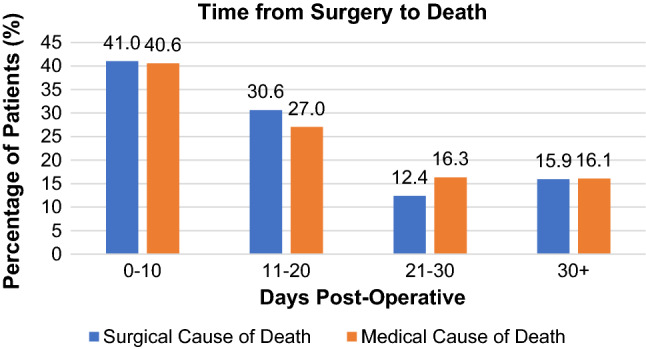
Comparison of the time from surgery to death in patients dying due to surgical causes versus medical causes

**Fig. 5:**
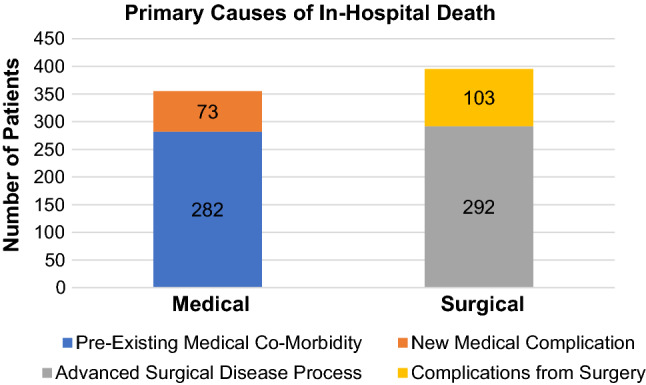
Primary causes of in-hospital death

The primary cause of in-hospital mortality was attributed to a surgical cause in 395 patients (52.7%) and a medical cause in 355 patients (47.3%). A comparison in the time from surgery to death between these two aetiologies is shown in Fig. 4. Amongst surgically related deaths, 292 patients (38.9%) died due to an advanced surgical pathology and 103 patients (13.7%) died due to complications from surgery. Amongst medically related deaths, 282 patients (37.6%) died due to complications arising from pre-existing medical co-morbidity and 73 patients (9.7%) died due to new medical complications unrelated to pre-existing conditions. This is shown in Fig. 5.

### Elective versus emergency patient deaths

Elective patients, when compared with emergency patients were predominantly of male sex (69.6% vs 50.6%, *p*<0.001) and of older age (76.1 vs 73.4 years, *p*=0.02). The elective group had lower ASA scores (*p*<0.001); however, had a similarly large proportion of patients determined to be at a ‘considerable’ and ‘expected’ risk of death pre-operatively (62.1 vs 65.1%, *p*=0.37). Both groups also demonstrated an equally high proportion of patients requiring post-operative ICU admission (86.4 vs 84.9%, *p*=0.62), with the time from surgery to death significantly longer in the elective group (16 vs 13 days, *p*=0.03). Both groups had a similar distribution of colorectal pathologies (*p*=0.14) and underwent similar resection types (*p*=0.99). There were no differences between both groups in the primary cause of death (*p*=0.20). A comparison between elective and emergency patients is shown in Table 4.

**Table 4:** Elective versus emergency patient deaths

	Elective *N*=171 (%)	Emergency *N*=579 (%)	Total	*P* Value
Age	76.1 (37.3–96.2)	73.4 (19.3–99.3)		0.02
*Sex*				
Male	119 (69.6)	293 (50.6)	338	<0.001
Female	52 (30.4)	286 (49.4)	412	
*ASA score*				
1	1 (0.6)	2 (0.4)	3	<0.001
2	26 (15.7)	25 (4.4)	51	
3	100 (60.2)	178 (31.5)	278	
4	35 (21.1)	277 (49.0)	312	
5	4 (2.4)	83 (14.7)	87	
*Co-morbidities*				
Cardiac	99 (63.1)	381 (68.0)	480	0.24
Respiratory	69 (43.9)	233 (41.6)	302	0.60
Renal	45 (28.7)	158 (28.2)	203	0.91
Hepatic	10 (6.4)	58 (10.4)	68	0.13
Neurological	37 (23.6)	83 (14.8)	120	0.01
Advanced malignancy	37 (23.6)	164 (29.3)	201	0.16
Diabetes	26 (16.6)	125 (22.3)	151	0.12
Obesity	31 (19.7)	86 (15.4)	117	0.19
Advanced age	100 (63.7)	302 (53.9)	402	0.03
*Risk of death on presentation*				
Minimal to moderate	64 (37.9)	196 (34.1)	260	0.37
Considerable and expected	105 (62.1)	379 (65.9)	484	
*ICU requirement*	146 (86.4)	488 (84.9)	634	0.62
*Time from surgery to death*	16 (0–126)	13 (0–113)		0.03
*Colorectal resection type*				
Right-sided colectomy	84 (49.1)	288 (49.7)	372	0.99
Left-sided colectomy	65 (38.0)	218 (37.7)	283	
Subtotal and total colectomy	22 (12.9)	73 (12.6)	95	
*Colorectal pathology*				
Malignancy	69 (40.4)	234 (40.7)	303	0.14
Ischaemia	26 (15.2)	124 (21.6)	150	
Inflammatory and Infective	33 (19.3)	106 (18.4)	139	
Obstructive	32 (18.7)	93 (16.2)	125	
Other	11 (6.4)	18 (3.1)	29	
*Primary cause of death*				
Medical				
Pre-existing medical co-morbidity	59 (35.1)	220 (38.1)	279	0.20
New medical complication	13 (7.7)	60 (10.4)	73	
Surgical				
Advanced surgical pathology	65 (38.7)	225 (39.0)	290	
Complications from surgery	31 (18.5)	72 (12.5)	103	

*ASA* American society of anaesthesiologists; *ICU* intensive care unit

## Discussion

This study critically examines the mortality outcome of patients who undergo colorectal resection. Our cohort had a mean ASA score of 3.6, and the majority underwent complex operations requiring post-operative ICU. Despite best care, we have demonstrated that advanced surgical pathology and medical co-morbidities both contribute to irreversible physiologic insult and result in early death after surgery. Together, this accounted for 77% of the deaths examined in the cohort. With 65% of patients quoted to have a high risk of death pre-operatively, it is arguable whether these patients should have had an operation in the first place. It is recognised that life-prolonging interventions frequently fail to align with a critically unwell patient’s personal goals, and often reduces quality of life [17].

Non-beneficial or futile surgery has been described in both physiological terms, where surgery would likely not result in any medical benefit, and controversially in qualitative terms, where surgery even if successful would likely result in an unacceptable quality of life [18]. The decision-making process to offer non-beneficial surgery has been described by Cooper et al. [19] to be complex and multifactorial. Surgeons often favour active interventions due to uncertainty on deciding whether the presenting illness represents a treatable condition or a terminal disease. Surgeons may also feel the need to do everything possible when the alternative is almost certainly death. In addition, many surgeons may feel reluctant to incorporate a palliative approach or discuss death, as they equate this with cessation of acute intervention, which is discordant with the surgical culture. In a time-constrained emergency scenario, it is also difficult to thoroughly evaluate the psychosocial wellbeing and physical function of a patient, and relate this in perspective to the acute situation impacting their physical health [20]. The ability of a patient to understand the gravity of their underlying illness is grossly limited when they are acutely unwell and has been shown to have a direct impact on their expectations for treatment. Patients who over-estimate their prognosis are more likely to prefer life-prolonging treatment over comfort-directed therapy [21]. Meanwhile, patients who undertake end-of-life discussions earlier in the course of illness and have an understanding of their prognosis are less likely to undergo burdensome treatments with a low likelihood of success [22].

In the modern era, healthcare decision-making has transitioned towards an integrated multi-disciplinary team (MDT) approach where all options are carefully considered. The decision to forego surgical intervention and opt for palliative measures does not denote failure, but in appropriate candidates represents a logical transfer to a better care pathway that is not only associated with higher levels of patient satisfaction, but also cost-effectiveness [23, 24]. Even when surgical intervention is chosen, pre-operative palliative care review initiated by a surgeon has also been associated with a marked reduction in operative related mortality (OR 0.27; 95% CI, 0.11–0.68), even after controlling for factors such as age and frailty [25]. This indicates that early palliative care involvement does not preclude active treatment in those who have opted for surgery, but may also serve to improve overall outcomes by assisting in goal-directed treatment of anticipated surgical complications if they occur [26].

Although elective surgery carries a significantly lower risk of post-operative mortality compared to emergency surgery [8–10], it is by no means innocuous. Few studies have compared the differences between elective and emergency patients in those who do ultimately die. Our results show that elective patients were predominantly of male sex and of older age with a greater co-morbidity burden. This is concordant with other studies which have consistently shown both male sex and advanced age to be independent predictors of 30 days mortality in the elective setting [11, 27, 28]. Interestingly, we have additionally demonstrated that elective patients who died were determined to be at an equally high risk of death pre-operatively compared to their emergency counterparts, and underwent similarly complex procedures that frequently necessitated post-operative ICU admission. In keeping with the aforementioned rationale on why surgeons may choose to operate on such patients, the absence of acute illness likely gives patients a higher level of expectations on their treatment and surgeons an even greater reason to do everything possible for the patient. The risks in operating on the elderly and severely co-morbid however, must not be masked under the pretences of elective surgery.

It is therefore clear that regardless of operation urgency, choosing the right patients for surgery through pre-operative patient prognostication is paramount to reduce unnecessary morbidity and mortality. Clinical judgement alone has been found to under predict mortality in very high-risk patients [29]. In colorectal surgery, the specialty-specific ColoRectal-Physiology and Operative Severity Score for the enUmeration of Mortality and Morbidity (CR-POSSUM) has been shown to have the greatest concordance with the true observed mortality rate compared to the original POSSUM score and its variant the Portsmouth POSSUM (P-POSSUM) score [30–33]. Leung et al. [30] reported that POSSUM, P-POSSUM and CR-POSSUM predicted a mortality rate of 13.5, 5 and 9.5%, respectively, in a cohort where the true observed mortality rate was 9%. In addition, Chiu et al. [34] and Al-Temimi et al. [35] have both identified that advanced age, sepsis and dependent functional status are independent risk factors associated with inevitable death following high-risk colorectal surgery. The consideration of such scores and risk factors may guide decision-making on the appropriateness of surgery when patients have been thought to have a high risk of death pre-operatively.

This study has also found that right hemicolectomy was the most common resection type resulting in in-hospital mortality. This is despite the fact that many surgeons believe it to be less technically demanding than left-sided resections, with anastomotic leak rates of right-sided anastomoses also lower compared to left-sided anastomoses [36]. Our findings are similar to Wilkins et al. [37] who reported that following colorectal cancer resection, right hemicolectomy was the greatest contributor to the number of deaths in the early post-operative period; and that 4.8% of right hemicolectomy patients died within 30 days post-operative compared to 1.9% of left hemicolectomy patients. These findings may be due to prolonged post-operative ileus (POI), which has been described to be highly associated with right-sided colonic resection [38]. Ileo-colic anastomoses have been demonstrated to have a significantly higher rate of prolonged ileus compared to colo-rectal anastomoses [39], potentially due to bacterial translocation and small intestinal bacterial overgrowth caused by ileo-caecal valve resection [40]. In addition, duodenal manipulation required in right-sided colonic mobilisation may induce a surge of adrenergic neuronal activity which additionally reduces gastric motility and may result in foregut ileus and ultimately aspiration [41, 42]. It may also be that the anastomotic leak rate is higher in right-sided resections but this was not a subject of investigation in this study and our data was not adept to examine this.

The limitation of this study was that although the qualitative surgical narrative allowed for a highly specific cause of death to be identified, confirmation bias could not be avoided as the SCF was written by the surgeon in hindsight. Their determination of the cause of death may have depended on their judgement of the events. Nonetheless in Australia, the cause of death is determined by the primary medical team and unless unexpected, it does not need to be verified by an independent reviewer. Whilst there was no chart review undertaken to verify the SCF, QASM data have previously been described as having a high concordance with medical records [43].

## Conclusions

This study comprehensively identifies the primary causes of in-hospital mortality following colorectal resection. As a group, these patients had significant co-morbidities and often presented with an advanced surgical pathology. Surgical and medical causes of death both contributed equally to the mortality burden.

## Supplementary Information

Below is the link to the electronic supplementary material.

Supplementary file1 (PDF 2204 KB)
