# Supplementary material for: Critical Analysis of the Causes of In-Hospital Mortality following Colorectal Resection: A Queensland Audit of Surgical Mortality (QASM) Registry Study
Source: World J Surg. 2022 Apr 4;46(7):1796–804. doi: 10.1007/s00268-022-06534-9 (PMC9174313; doi:10.1007/s00268-022-06534-9)
Supplement: Supplementary file 1 — Supplementary file1 (PDF 2204 KB) [file 268_2022_6534_MOESM1_ESM.pdf]

APPENDIX

S1: The Queensland Audit of Surgical Mortality (QASM) Surgical Case Form

Surgical Case Form

Important

1. **Please do not destroy this form**
2. **Please do not copy this form**
3. **Please return this form to the audit office (in reply paid envelope provided)**

By submitting this form to the Mortality Audit, I agree that Australian and New Zealand Audit of Surgical Mortality (ANZASM) may inform the Professional Standards Department of my involvement with the surgical mortality audit, to confirm my compliance with Continuing Professional Development (CPD) requirements.

Exclusion for terminal patients

Please complete this section for all patients

Was terminal care planned for this patient ***prior*** *to* ***<u>or on</u>*** *admission?*

☐ YES

If **YES** please describe the terminal condition:

....

....

☐ NO (Go to page 2 and complete ALL questions on this form)

If **YES**, was an operation performed on this terminal care patient?

☐ YES - Go to page 2 and complete ALL questions on this form

☐ NO (this patient is EXCLUDED from the audit; do NOT complete this form)

Return this form to the audit office.

All identifiers will be removed by the Audit office on receipt of this completed form

Study ID: ☐

Gender: ☐

DOB: ☐

Admission Date: ☐

Date of Death: ☐

Specialty: ☐

Hospital ID: ☐

Patient name: ☐

UMRN: ☐

Hospital: ☐

Consultant surgeon: ☐

Name of any Surgeon(s)/Trainee(s) to whom individual feedback should be sent:

☐

Anaesthetist(s) – please name:

☐

Study Number

1

Status of surgeon completing form:

- Consultant ☐
- Fellow ☐
- International Medical Graduate ☐
- SET trainee ☐
- Service Registrar ☐
- GP surgeon ☐
- Consultant confirms they have agreed to contents of the form YES ☐

Specialty of consultant surgeon in charge of patient:

- General ☐
- Vascular ☐
- Urology ☐
- Neurosurgery ☐
- Orthopaedics ☐
- Otolaryngology Head and Neck ☐
- Ophthalmology ☐
- Paediatrics ☐
- Obstetrics and Gynaecology ☐
- Plastic ☐
- Oral/Maxillofacial ☐
- Cardiothoracic ☐
- Other (specifiy) ☐ ....

2

Patient Age...........

Patient Sex: Male ☐ Female ☐ **Hospital Status:** Private ☐ Public ☐ Co-Located ☐

Aboriginal/ Torres Strait Islander descent? Yes ☐ No ☐

Admission Type: Elective ☐ Emergency ☐ **Patient Status:** Private ☐ Public ☐ Veteran ☐

Patient admitted by a surgeon? Yes ☐ No ☐

3

Main surgical diagnosis on admission *(as suspected by clinicians after initial assessment)*

....

....

Confirmed main surgical diagnosis *(taking into account test results, operations, post mortem etc)*

....

....

Final cause of death *(taking all information into account, including post mortem)*

....

....

4

Were there significant co-existing factors increasing risk of death? Yes ☐ No ☐ *(Tick all that apply)*

- Cardiovascular ☐
- Hepatic ☐
- Diabetes ☐
- Age ☐
- Respiratory ☐
- Neurological ☐
- Obesity ☐
- Advanced malignancy ☐
- Renal ☐
- Other (specify) ☐ ....

5

ASA 1 - A normal healthy patient ☐

ASA 2 - A patient with mild systemic disease ☐

ASA 3 – A patient with severe systemic disease which limits activity, but is not incapacitating ☐

ASA 4 – A patient with an incapacitating systemic disease that is a constant threat to life ☐

ASA 5 – A moribund patient who is not expected to survive 24 hrs, with or without an operation ☐

ASA 6 – A brain-dead patient for organ donation ☐

6

Was the patient **transferred pre-op**? Yes ☐ No ☐ If **NO**, go to Q7

Transferred from hospital ....

Transferred to hospital.. Distance (km)................

Was there a delay in transfer? Yes ☐ No ☐

Was the transfer appropriate? Yes ☐ No ☐

Was the level of care appropriate? Yes ☐ No ☐

Was there sufficient clinical information? Yes ☐ No ☐

7

Was there a **pre-op delay in** confirmation of main surgical diagnosis? Yes ☐ No ☐

If **NO**, go to Q8a

Was the delay associated with: GP ☐ Medical Unit ☐ Surgical Unit ☐ Other (specify) ☐

Was this due to: *(tick all that apply)* ....

Inexperience of staff ☐ Misinterpretation of results ☐ Unavoidable factors ☐

Failure to do correct test ☐ Results not seen ☐ Other (specify) ☐

....

8a

Was this patient **treated** in a critical care unit (ICU or HDU) during this admission? Yes ☐ (go to Q8b) No ☐ (continue)

Should this patient have been provided critical care in:

Intensive Care Unit (ICU)? Yes ☐ (continue) No ☐ (go to Q9)

High Dependency Unit (HDU)? Yes ☐ (continue) No ☐ (go to Q9)

Why did this patient not receive critical care? (tick all that apply and then go to Q9)

No ICU/ HDU bed available ☐

Admission refused by critical care staff ☐

No critical care unit in the hospital ☐

Active decision not to refer to critical care unit ☐

Not applicable ☐

8b

Was the surgical team satisfied with the critical care unit management of this patient? Yes ☐ (go to Q9) No ☐ (specify reasons below)

Specify..

....

....

9 Please describe the course to death (or attach report)
(*use back of form if required*)

10 Was an operation performed within 30 days of death or during the last admission? Yes ☐ No ☐

If **YES**, go to Q11. If **NO**: *(tick as necessary)*

It was not a surgical problem ☐

Active decision not to treat or operate ☐ ⟶ Was this a consultant's decision? Yes ☐ No ☐

Patient/family refused operation ☐

Rapid Death ☐

*If **NO** operation was performed, please go to Q18*

11 Surgeon's view (before any surgery) of overall risk of death

Minimal ☐ Small ☐ Moderate ☐ Considerable ☐ Expected ☐

12 Description of operation(s) (including relevant radiological or endoscopic procedures)

Operation (1) Date .... /......... / .... Start time............:............. (24hr clock) Estimated length (hours) of operation ☐

....

....

....

....

Operation (2) Date .... /......... / .... Start time............:............. (24hr clock) Estimated length (hours) of operation ☐

....

....

....

....

Operation (3) Date .... /......... / .... Start time............:............. (24hr clock) Estimated length (hours) of operation ☐

....

....

....

....

13 **Timing of operation**

	1st Op	2nd Op	3rd Op
Elective	☐	☐	☐
Immediate (< 2 hours)	☐	☐	☐
Emergency (< 24 hours)	☐	☐	☐
Scheduled emergency (> 24 hours after admission)	☐	☐	☐

14

Was there a consultant anaesthetist present at the operation?

	1st Op	2nd Op	3rd Op
Yes			
No			

Was the operation abandoned on finding a terminal situation?

	1st Op	2nd Op	3rd Op
Yes			
No			
N/A			

15

Grades of surgeons making decisions, operating, assisting and present in theatre

	**1st Op**				**2nd Op**				**3rd Op**			
	Decide	Operate	Assist	In Theatre	Decide	Operate	Assist	In Theatre	Decide	Operate	Assist	In Theatre
Consultant												
Fellow												
International Medical Graduate												
SET trainee												
Service Registrar												
GP surgeon												
None												

16

Was there a definable **post-operative** complication? Yes ☐ No ☐ *If **NO**, go to Q17*

Surgical **complications** relating to present admission *(please tick all that apply)*

Anastomotic leak ☐ site ⟶ Oesophageal ☐ Pancreas/biliary ☐ Colorectal ☐

Gastric ☐ Small Bowel ☐

Procedure related sepsis ☐ Tissue ischaemia ☐

Significant post-op bleeding ☐ Vascular graft occlusion ☐

Endoscopic perforation ☐ Other (specify) ☐

....

....

....

....

Was there a **delay in recognising** post-operative complications? Yes ☐ No ☐

17

Was there an anaesthetic component to this death? Yes ☐ No ☐ Possibly ☐

Was death within 48 hours of last anaesthetic? Yes ☐ No ☐ Don't know ☐

18

Was a post-mortem examination performed?

Yes – hospital ☐ Yes – coroner ☐ No ☐ Refused ☐ Unknown ☐

19

Was DVT prophylaxis used during this admission? Yes ☐ No ☐

If **YES** (tick all that apply)

Heparin (any form) ☐ Aspirin ☐ TED Stockings ☐

Warfarin ☐ Sequential compression device ☐ Other (specify) ☐

....

If ***NO***, state reasons: Not appropriate ☐ Active decision to withhold ☐ Not considered ☐

*and please comment on why **NOT** used* ....

....

....

....

20

	Yes	No	Unknown
Was there an **unplanned return** to theatre?	☐	☐	☐
Was there an **unplanned admission** to a critical care unit?	☐	☐	☐
Was there an **unplanned readmission** within 30 days of surgery?	☐	☐	☐
Was **fluid balance** an issue in this case?	☐	☐	☐
Was there an issue with **communication** at any stage?	☐	☐	☐

21a

Did this patient die with a **clinically-significant infection**? Yes ☐ (continue) No ☐ (go to Q23)

Was this infection acquired: before this admission ☐ (go to Q21b) or during this admission ☐ (continue)

If acquired **during** this admission, was the infection: acquired pre-operatively ☐ or a surgical-site infection ☐

or acquired post-operatively ☐ or other invasive-site infection ☐

21b

Was the **infection**: pneumonia ☐ intra-abdominal sepsis ☐ septicaemia ☐ other source ☐

Was the infective organism identified? Yes ☐ No ☐

If yes, what was the organism? ....

....

....

Was there a delay in treatment of the infection? Yes ☐ No ☐

22

Was the **antibiotic regimen** appropriate? Yes ☐ No ☐ Unknown ☐ Not applicable ☐

23 Do you consider **management** could have been **improved** in the following areas?

Area	Yes	No	N/A	Area	Yes	No	N/A
Pre-operative management/ preparation	☐	☐	☐	Intra-operative/technical management of surgery	☐	☐	☐
Decision to operate at all	☐	☐	☐	Grade/experience of surgeon deciding	☐	☐	☐
Choice of operation	☐	☐	☐	Grade/experience of surgeon operating	☐	☐	☐
Timing of operation *(too late, too soon, wrong time of day)*	☐	☐	☐	Post operative care	☐	☐	☐

24a An area for CONSIDERATION is where the clinician believes areas of care COULD have been IMPROVED or DIFFERENT, but recognises that it may be an area of debate.
An area of CONCERN is where the clinician believes that areas of care SHOULD have been better.
An ADVERSE EVENT is an unintended injury caused by medical management rather than by disease process, which is sufficiently serious to lead to prolonged hospitalisation or to temporary or permanent impairment or disability of the patient at the time of discharge, or which contributes to or causes death.

Were there any issues in the management of this patient? Yes ☐ (please describe below) No ☐ (please go to Q25)

24b ***Important:*** *please describe the 3 most significant events and list any other events.*

i). (please describe most significant event) ....

....

Area of:		Which:		Was the event preventable?		Associated with?	
Consideration	☐	Made no difference to outcome	☐	Definitely	☐	Audited Surgical team	☐
Concern	☐	May have contributed to death	☐	Probably	☐	Another Clinical team	☐
Adverse event	☐	Caused death of patient who would otherwise be expected to survive	☐	Probably not	☐	Hospital	☐
				Definitely not	☐	Other (please specify)	☐

ii). (please describe the second most significant event). ....

....

Area of:		Which:		Was the event preventable?		Associated with?	
Consideration	☐	Made no difference to outcome	☐	Definitely	☐	Audited Surgical team	☐
Concern	☐	May have contributed to death	☐	Probably	☐	Another Clinical team	☐
Adverse event	☐	Caused death of patient who would otherwise be expected to survive	☐	Probably not	☐	Hospital	☐
				Definitely not	☐	Other (please specify)	☐

iii). (please describe the third most significant event). ....

....

Area of:		Which:		Was the event preventable?		Associated with?	
Consideration	☐	Made no difference to outcome	☐	Definitely	☐	Audited Surgical team	☐
Concern	☐	May have contributed to death	☐	Probably	☐	Another Clinical team	☐
Adverse event	☐	Caused death of patient who would otherwise be expected to survive	☐	Probably not	☐	Hospital	☐
				Definitely not	☐	Other (please specify)	☐

24c **List other events**..

....

....

....

....

25 **In retrospect, would you have done anything differently?** Yes ☐ No ☐

*If **YES**, please specify*..

....

....

....

....

....

....

....

....

....

26 Was **trauma** involved? Yes ☐ (continue) No ☐ unknown ☐

(a) Was the trauma the result of a **fall**?

Yes ☐ (continue)

No ☐ (go to (b))

If yes, please indicate:

- fall at home ☐
- fall in a care facility ☐
- fall in hospital ☐
- unknown ☐
- other* (sport/recreation/farm/work) ☐

specify*..

....

....

(b) Was the trauma the result of a **road traffic accident**?

Yes ☐ (continue)

No ☐ (go to (c))

If yes, please indicate:

- motor vehicle accident ☐
- motor bike accident ☐
- bicycle accident ☐
- pedestrian accident ☐
- unknown ☐
- other* ☐

specify*..

....

....

(c) Was the trauma the result of **violence**?

Yes ☐ (continue)

No ☐

If yes, please indicate:

- domestic violence ☐
- public violence ☐
- self-inflicted violence ☐
- unknown ☐
- other* ☐

specify*..

....

....

Additional comments

ROYAL AUSTRALASIAN COLLEGE OF SURGEONS

THANK YOU

FOR OFFICE USE

Date sent ....

Date received ....

Date coded / entered ....

Entered by .... Checked by ....

Date sent to FLA..........

Date received from FLA..........

No further action ☐

For assessment ☐

Medical records requested ....

Medical records received..........

Date sent to SLA..........

Date received from SLA..........

Case completed..........

Coding: Yes=1, No=2, Don't know=3

S2: Rural, Remote and Metropolitan Areas (RRMA) Classification

**Zone**	**RRMA Classification**	**Definition**
Metropolitan (M)	M1	Capital Cities
	M2	Other Metropolitan Centres<br>(Urban centre population ≥100,000)
Rural (R)	R1	Large Rural Centres<br>(Urban centre population 25,000-99,999)
	R2	Small Rural Centres<br>(Urban centre population 10,000-24,999)
	R3	Other Rural Areas<br>(Urban centre population <10,000)

DPIE (Department of Primary Industries and Energy) & DHSH (Department of Human Services and Health) 1994. Rural, Remote and Metropolitan Areas classification. 1991 Census edition. Canberra: Australian Government Publishing Service.
